# Precise Identification and Analysis of Maize Germplasm Resistance to Ear Rot Caused by Six *Fusarium* Species

**DOI:** 10.3390/plants14152280

**Published:** 2025-07-24

**Authors:** Shuai Li, Lihong Zhu, Yongxiang Li, Yaxuan Guo, Yuhang Zhang, Chaosong Huang, Wenqi Wu, Suli Sun, Zixiang Cheng, Canxing Duan

**Affiliations:** State Key Laboratory of Crop Gene Resources and Breeding, Institute of Crop Sciences, Chinese Academy of Agricultural Sciences, Beijing 100081, China; lstheone0625@163.com (S.L.); zlh199706220722@163.com (L.Z.);

**Keywords:** maize germplasm, *Fusarium* species, ear rot, resistance, precise identification

## Abstract

Maize (*Zea may* L.) is one of the most important crops worldwide, but ear rot poses a significant threat to its production. Diverse pathogens cause ear rot in China, with *Fusarium* spp. being predominant, especially *Fusarium graminearum* and *Fusarium verticillioides*. Current methods for the control of ear rot are limited, making the use of resistant germplasm resources an effective and economical management strategy. Earlier research focused on resistance to Fusarium ear rot (FER; caused by *F. verticillioides*) and Gibberella ear rot (GER; caused by *F. graminearum*), but assessing maize resistance to multiple major *Fusarium* spp. is critical in ensuring maize production. Thus, the resistance of 343 maize germplasm resources to ear rot caused by six *Fusarium* spp. (*F. verticillioides*, *F. graminearum*, *F. proliferatum*, *F. meridionale*, *F. subglutinans*, and *F. temperatum*) was evaluated in this study. Over three years, 69 and 77 lines resistant to six and five ear rot diseases, respectively, and 139 lines resistant to both FER and GER were identified. Moreover, the 343 germplasm resources were divided into eight heterotic groups, of which PH4CV was the most resistant one, whereas NSS and Pioneer Female were the least resistant ones. These findings provide a basis for the development of maize cultivars with broad-spectrum ear rot resistance.

## 1. Introduction

Maize (*Zea mays* L.) is one of the main crops in terms of both planting area and yield. With a total production volume of 12.14 billion tons and a planting area of 161.7 million hectares in 2024, according to USDA data, maize is the most significant grain crop worldwide, with substantial contributions to the global food supply and agriculture-based economies. It has critical roles associated with feed and food production and industrial processing, thereby directly affecting food security and sustainable development [1].

Maize ear rot represents the most severe disease complex in maize-growing regions worldwide [2], primarily infecting kernels, along with significant yield and quality losses. This complex encompasses distinct diseases, such as Fusarium ear rot (FER), Gibberella ear rot (GER), and Aspergillus ear rot (AER), each caused by specific fungal pathogens. In China, its prevalence is severe, with infection rates generally ranging from 10% to 20%, and they can reach 40–50% during epidemics. Continuous rainy conditions during the grain-filling stage exacerbate maize ear rot outbreaks, leading to yield losses of 30–40%. In severe cases, the infection rates can reach 40–100% for some susceptible varieties [3]. Globally, more than 70 species have been identified as causal agents of maize ear rot, including *Fusarium* spp., *Aspergillus* spp., *Penicillium* spp., *Trichoderma* spp., *Stenocarpella maydis*, *Trichoderma atroviride*, *Sarocladium zeae*, *Lecanicillium lecanii*, and *Trichothecium roseum*. These fungi contribute significantly to maize ear rot severity and mycotoxin contamination, with detrimental effects on both crop yields and safety. Among these species, *Fusarium* spp. are the most prominent pathogens worldwide [4]. Moreover, they are primarily transmitted through soil, plant residues, or infected seeds, often overwintering as mycelia or spores. Maize ear rot spreads via soil, insect vectors, or rainwater, with the field temperature and humidity playing key roles in disease development. Favorable temperatures and high humidity are particularly conducive to the outbreak and spread of this disease [5,6,7].

Pathogens causing maize ear rot can decrease maize yields, while also producing various harmful toxins that significantly affect grain quality. *Fusarium* spp., the primary pathogens, produce different toxins during infection, with deoxynivalenol (DON) and fumonisins being the most common. For example, *F. verticillioides* predominantly produces fumonisins, which are highly toxic to animals, causing delayed growth, disease, or even death. In humans, fumonisins are linked to esophageal, liver, and stomach cancers [8]. The *F. graminearum* species complex primarily produces zearalenone (ZEN), nivalenol, and DON. ZEN exposure can cause multiple symptoms, including weakness, dizziness, diarrhea, and severe disruptions to the central nervous system. DON has highly cytotoxic and immunosuppressive effects, posing significant risks to human and livestock health. Ingesting DON-contaminated food can lead to acute poisoning symptoms, including vomiting, diarrhea, fever, unsteady gait, and delayed reactions, with severe cases causing hematopoietic system damage and death [9,10].

In China, *Fusarium* spp. are the primary pathogens causing maize ear rot, with significant diversity in pathogens and regional distributions [11,12]. Duan collected 239 maize ear rot samples from 18 provinces in 2009–2014, revealing that *F. verticillioides* and *F. graminearum* represented 95.1% of the identified *Fusarium* isolates. Other *Fusarium* spp. included *F. culmorum*, *F. oxysporum*, *F. proliferatum*, *F. subglutinans*, and *F. solani* [9]. In a previous study involving 14 provinces, Ren identified *Fusarium* spp. as the dominant pathogens, with *F. verticillioides* and *F. graminearum* being the most common. In addition to *F. verticillioides* and *F. graminearum*, the other isolated *Fusarium* spp. included *F. proliferatum*, *F. oxysporum*, *F. subglutinans*, *F. culmorum*, *F. solani*, and *F. semitectum*, which were sorted according to the number of detected isolates [13]. In a study conducted by Zhou et al. from 2014 to 2015 in Chongqing and the surrounding areas, 10 *Fusarium* spp. were isolated from maize ear rot samples (*F. verticillioides*, *F. proliferatum*, *F. graminearum* species complex, *F. oxysporum* species complex, *F. fujikuroi*, *F. equiseti*, *F. culmorum*, *F. incarnatum*, *F. kyushuense*, and *F. solani*); the dominant pathogens were *F. verticillioides*, the *F. graminearum* species complex, and *F. proliferatum* [14]. In a study by Sun et al., *Fusarium* spp. were isolated from maize ear rot samples collected in Hainan in 2016; five species were identified, including *F. verticillioides*, *F. subglutinans*, *F. equiseti*, and *F. andiyazi* [15]. In 2018, Wang collected 143 typical ear rot samples from 21 maize-producing counties in Heilongjiang. From 200 single-spore isolates, 12 *Fusarium* spp. were identified. The species, listed in descending order of frequency, were *F. graminearum*, *F. verticillioides*, *F. subglutinans*, *F. proliferatum*, *F. boothii*, *F. temperatum*, *F. andiyazi*, *F. incarnatum*, *F. sporotrichioides*, *F. poae*, *F. commune*, and *F. asiaticum* [16]. Chai et al. collected 254 maize ear rot samples across Jilin Province during 2020–2021. Sixteen pathogenic *Fusarium* species were isolated, with *F. verticillioides* and *F. graminearum* exhibiting the highest isolation frequencies. Subdominant species included *F. proliferatum*, *F. boothii*, *F. subglutinans*, *F. temperatum*, *F. asiaticum*, *F. chlamydosporum*, *F. fujikuroi*, *F. equiseti*, and *F. subglutinans* (in descending order of prevalence) [17,18]. Wang et al. collected 36 maize ear rot samples in Gansu Province in 2020–2021, isolating eight pathogenic *Fusarium* species. Their isolation frequencies were as follows: *F. verticillioides* (68.49%), *F. proliferatum* (19.18%), *F. subglutinans* (6.85%), *F. boothii* (2.40%), *F. graminearum* (1.71%), *F. temperatum* (0.69%), *F. poae* (0.34%), and *F. andiyazi* (0.34%) [19].

The geographic distribution and ecological dominance of the main *Fusarium* species in China are presented in Table 1. *F. verticillioides* and *F. graminearum* are the dominant pathogens causing maize ear rot across China [20], being more prevalent in the northeastern regions than in other areas. *F. subglutinans* and *F. temperatum* are more commonly found in Northern China, while there is considerable diversity in the *Fusarium* spp. in the central and southern regions. *F. proliferatum* is mainly found in central provinces, whereas the *F. graminearum* species complex is often isolated in southern regions [21]. Additionally, *F. proliferatum*, *F. subglutinans*, and *F. temperatum* have been detected in various regions and are becoming secondary dominant pathogens. Because of the diversity of these pathogens, crop rotations and fungicide applications have had limited effects in preventing maize ear rot [22], highlighting the importance of strengthening maize resistance through the selection of stable, multi-resistant germplasm, which is key for long-term disease management and breeding.

From 2006 to 2009, Duan et al. evaluated the resistance of 836 superior maize resources to FER, identifying 5 highly resistant, 71 resistant, and 388 moderately resistant germplasm resources [23]. Between 2006 and 2012, Duan et al. evaluated 1647 maize germplasm resources for resistance to FER, identifying 27 highly resistant, 352 resistant, and 784 moderately resistant lines [24]. Between 2009 and 2011, Guo et al. used a natural field infection method to assess maize resistance to Fusarium ear rot and identified 74 highly resistant, 55 resistant, and 275 moderately resistant maize lines [25]. From 2018 to 2020, Han et al. analyzed the resistance of 10,524 maize germplasm resources to FER and GER, identifying 191 highly resistant lines, which were evaluated further, ultimately revealing 59 lines with stable resistance [26]. Zhang et al. screened 44 maize inbred lines for resistance to both FER and GER and identified three highly resistant lines [27]. Zhao et al. precisely evaluated 48 maize inbred lines and selected five lines resistant to GER [28]. In 2018–2020, Duan et al. examined 690 representative maize germplasm resources resistant to FER, identifying 35 germplasm resources with stable resistance to FER [29]. Xia et al. screened 346 maize inbred lines, identifying 45 lines that were at least moderately resistant to both FER and GER [30]. The dominant pathogens in different regions can change over time [14,31]. To address the potential risks from shifts in predominant pathogens, in addition to focusing on FER and GER, attention should be paid to ear rot caused by *F. proliferatum*, *F. subglutinans*, and *F. temperatum*, which are emerging threats to maize cultivation [32]. A shift from *F. culmorum* to *F. graminearum* and *F. verticillioides* in Heilongjiang is an example of a change in the dominant pathogens over time. Additionally, multiple dominant pathogens may coexist within the same province. For example, in Shandong, *F. verticillioides* is widely distributed, but *F. poae* and *F. graminearum* are major pathogens in the central, eastern, and southwestern regions [33].

Current evaluations of maize germplasm resistance to ear rot are largely focused on resistance to *F. verticillioides* and *F. graminearum*, leaving broad-spectrum resistance to multi-Fusarium systems critically underexplored. Although *F. proliferatum*, *F. moniliforme*, *F. subglutinans*, and *F. temperatum* are also primary pathogenic fungi, their geographic distribution varies significantly across regions. Therefore, there is a critical need for analyses of resistance to multiple *Fusarium* spp. to ensure safe maize production. To address this gap, we conducted China’s first systematic screening of 343 maize inbred lines against six economically impactful *Fusarium* species (*F. verticillioides*, *F. graminearum*, *F. proliferatum*, *F. subglutinans*, *F. temperatum*, and *F. meridionale*) across three years (2022–2024), with the objectives of identifying multi-resistant germplasm and quantifying pathogen correlation networks.

## 2. Results

From 2022 to 2024, 343 maize inbred lines were evaluated for resistance to ear rot caused by six *Fusarium* species. The resistance level was assessed in visual and machine-assisted surveys. With the exception of 2023, where extreme weather events resulted in significantly lower disease incidence, all other years exhibited epidemiologically effective infection pressure for each *Fusarium* species (Table 2).

**Figure 1:**
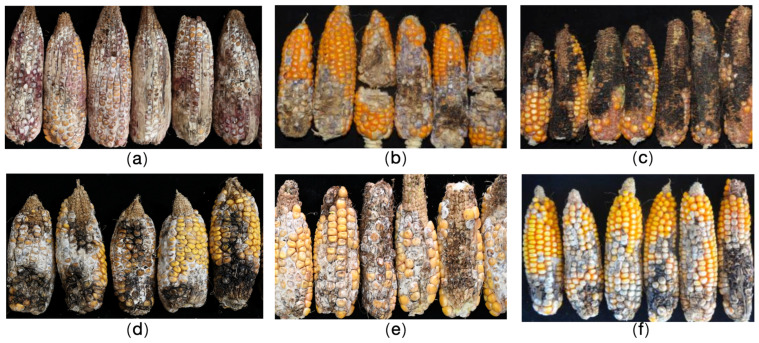
Symptoms of maize ear rot caused by *Fusarium* spp. in the field. (**a**) Ear rot caused by *F. graminearum*; (**b**) ear rot caused by *F. temperatum*; (**c**) ear rot caused by *F. subglutinans*; (**d**) ear rot caused by *F. proliferatum*; (**e**) ear rot caused by *F. verticillioides*; (**f**) ear rot caused by *F. meridionale*. Scale bar: 5 cm.

Figure 1 illustrates the typical field symptoms of ear rot caused by the six species considered in this study. Maize infected with *F. graminearum* developed purplish red lesions on ear tissues. In contrast, an infection by *F. temperatum* resulted in pale blue lesions, whereas an infection by *F. subglutinans* mostly induced black necrotic lesions on infected ears. The main symptoms of infections by the remaining three *Fusarium* spp. were white or whitish lesions at infection sites. These distinct disease symptoms provide critical diagnostic markers for the identification of ear rot pathogens under field conditions.

**Figure 2:**
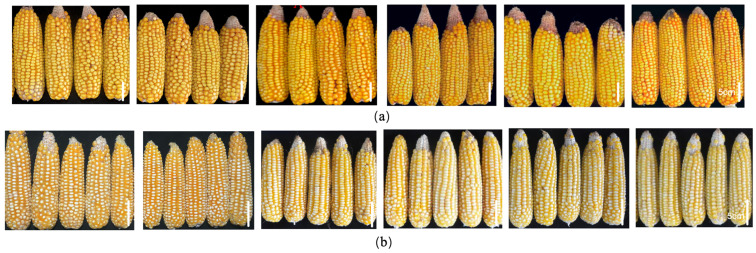
Selected materials highly resistant to six ear rot pathogens. (**a**) K21HZD2596; (**b**) K21HZD5342. From left to right: maize ears infected with *F. graminearum*, *F. proliferatum*, *F. subglutinans*, *F. temperatum*, *F. verticillioides*, and *F. meridionale*. Scale bar: 5 cm.

Of the 343 germplasm resources evaluated over 3 years, 69 and 77 lines were resistant to six and five ear rot types caused by *Fusarium* species, respectively, whereas 139 lines were resistant to both FER and GER. Some lines were highly resistant to all six ear rot diseases caused by *Fusarium* species, including K21HZD2610, K21HZD0611, K21HZD2374, K21HZD6018, K21HZD0057, K21HZD2879, K21HZD2596, and K21HZD5342. Selected highly resistant materials are presented in Figure 2.

**Figure 3:**
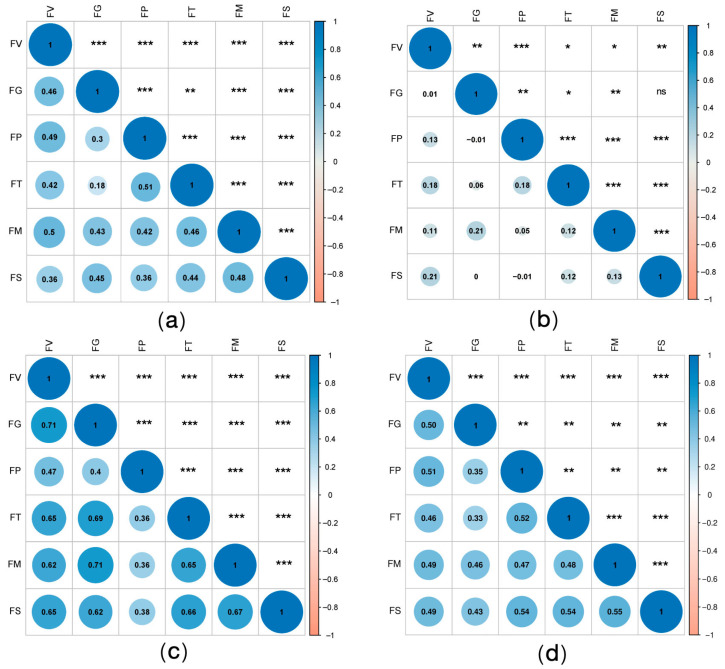
Heatmap of correlations between the resistance of tested materials to six ear rot pathogens between 2022 and 2024. (**a**) 2022; (**b**) 2023; (**c**) 2024; (**d**) 2022–2024. (FV: FER, FG: GER, FP: *F. proliferatum* ear rot, FT: *F. temperatum* ear rot, FM: *F. meridionale* ear rot, FS: *F. subglutinans* ear rot), data markers indicate statistical significance (*: *p* < 0.05; **: *p* < 0.01; ***: *p* < 0.001).

Correlations among the resistances of the tested germplasm resources to six ear rot pathogens exhibited marked interannual variability between 2022 and 2024. In 2022, the resistance correlations (*r*-values) ranged from 0.18 to 0.51 (Figure 3a), with the *r*-values for most pairwise comparisons exceeding 0.4, indicating moderate correlations. Exceptions included the resistance to GER, which had a relatively weak association with the resistance to ear rot caused by *F. temperatum* or *F. proliferatum*. However, in 2023, the *r*-values decreased to −0.01–0.21 (Figure 3b), likely because of extreme environmental stressors, including sustained high temperatures (>40 °C) and unseasonal late rainfall, which suppressed uniform disease development and obscured intrinsic resistance relationships. By 2024, the correlations rebounded robustly, with *r*-values between 0.36 and 0.71 (Figure 3c). Resistance correlations increased significantly, with the *r*-values for all comparisons, except those involving ear rot caused by *F. proliferatum*, exceeding 0.62. Notably, GER resistance was highly correlated with the resistance to FER and ear rot caused by *F. subglutinans*, suggesting that there may be shared resistance mechanisms under certain environmental conditions. A heatmap of the correlations between the resistance of the tested materials to the six ear rot pathogens from 2022 to 2024 revealed a general lack of strong correlations among the resistance to most *Fusarium* spp. (*r*-values of 0.16 to 0.54) (Figure 3d). However, the *r*-value for the resistance of the tested materials to FER and ear rot caused by *F. temperatum* or *F. proliferatum* was approximately 0.5, indicating a moderate correlation.

The evaluation of the resistance to the six ear rot pathogens indicated that 51.99% of the tested germplasm resources were moderately resistant, 28.47% were susceptible, 16.52% were resistant, 2.24% were highly susceptible, and only 0.78% were highly resistant. Thus, most of the tested materials were moderately resistant to the six ear rot pathogens, whereas highly susceptible or highly resistant accessions were relatively rare.

**Figure 4:**
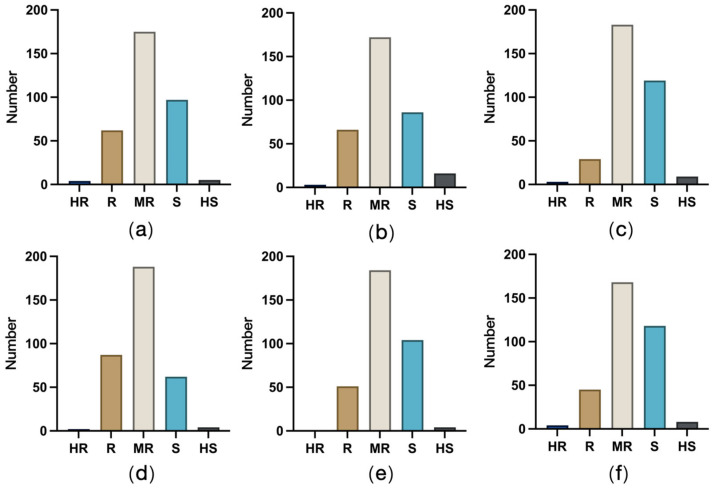
Distribution of germplasm resistance to ear rot caused by six pathogens. (**a**) FER; (**b**) GER; (**c**) ear rot caused by *F. proliferatum*; (**d**) ear rot caused by *F. temperatum*; (**e**) ear rot caused by *F. meridionale*; (**f**) ear rot caused by *F. subglutinans*.

According to an analysis of germplasm resistance to FER and GER, four and three accessions were highly resistant, 62 and 66 were resistant, and 175 and 172 were moderately resistant to FER and GER, respectively. Five accessions were highly susceptible to FER (Figure 4a), but 16 accessions were highly susceptible to GER (Figure 4b), representing the highest number of accessions highly susceptible to one of the six ear rot pathogens included in this study. The number of susceptible accessions was the highest for ear rot caused by *F. proliferatum* (119 accessions; 34.6%) (Figure 4c), followed by ear rot caused by *F. subglutinans* (118 accessions; 34.3%) (Figure 4f). In terms of the smallest number of resistant accessions, only 29 accessions (8.4%) were resistant to ear rot caused by *F. proliferatum*, which was in contrast to the 45 accessions resistant to ear rot caused by *F. subglutinans*. A total of 66 (19.1%) accessions were highly susceptible or susceptible to ear rot caused by *F. temperatum* (Figure 4d), which was smaller than the number of accessions highly susceptible or susceptible to ear rot caused by the other pathogens. No accession was highly resistant to ear rot caused by *F. meridionale* (Figure 4e). Overall, the tested accessions were most resistant to ear rot caused by *F. temperatum*, followed by FER and GER. Accessions resistant to ear rot caused by *F. subglutinans* or *F. meridionale* were generally moderately resistant. The poorest resistance performance was observed for ear rot caused by *F. proliferatum*.

An analysis of variance (ANOVA) across three years revealed significant genotypic variance (*p*-value < 0.05) for resistance to all six *Fusarium* species, indicating stable and heritable differences in ear rot resistance among the 343 maize inbred lines. The largest genotypic variances (genotypic variance > 1.6) were observed for GER and FER, suggesting that these two species are the most suitable targets for resistance breeding (Table 3).

In contrast, *F. proliferatum* ear rot, *F. temperatum* ear rot, *F. meridionale* ear rot, and *F. subglutinans* ear rot showed strong genotype-by-environment interactions, with G × E effects accounting for over 32% of the total phenotypic variation. This indicates that resistance to these species is highly influenced by environmental factors, particularly in the case of *F. temperatum* ear rot, which exhibited the strongest environmental dependency. GER and FER had comparatively lower G × E effects (<21%), reflecting more stable resistance performance across different environments (Table 4).

**Figure 5:**
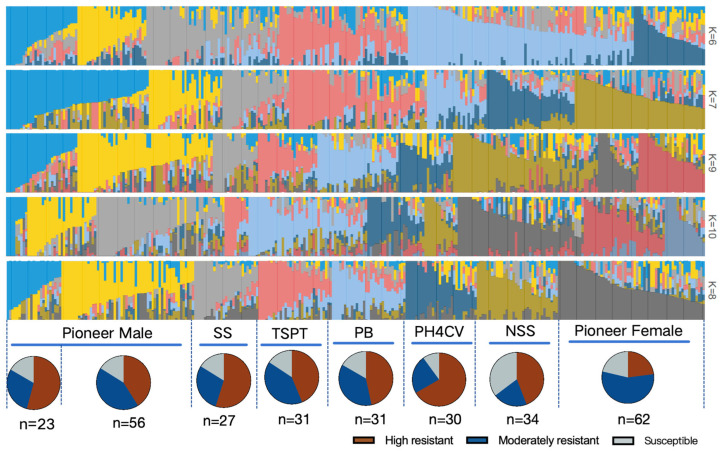
Heterotic group classification of maize germplasm resources. NSS (34 accessions); SS (27 accessions); TSPT (31 accessions); PB (31 accessions); and PH4CV (30 accessions). According to the observed resistance to six ear rot pathogens, the germplasm resources were categorized as follows: highly resistant accessions (resistant to five or six *Fusarium* species), moderately resistant accessions (resistant to two to four *Fusarium* species), and susceptible accessions (susceptible to five or six *Fusarium* species).

STRUCTURE 2.2 was used for the heterotic group classification of 294 of 343 accessions. An analysis of the population structure revealed that the ΔK value was the highest when K was 8, indicative of eight major heterotic groups (Figure 5). These groups were as follows: Pioneer Male (79 accessions), subdivided into Pioneer Male A (23 accessions) and Pioneer Male B (56 accessions), and Pioneer Female (62 accessions).

**Figure 6:**
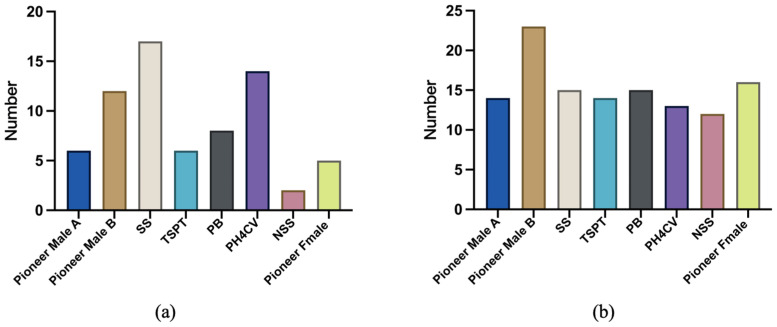
Classification of maize germplasm resources on the basis of different hierarchies. (**a**) Accessions resistant to six ear rot pathogens; (**b**) accessions resistant to FER and GER.

The SS group had the most accessions resistant to all six *Fusarium* species (17 accessions), followed by the PH4CV group (14 accessions) and the Pioneer Male B group (12 accessions). By contrast, the NSS group consisted of only two accessions resistant to all six *Fusarium* species (Figure 6a). The Pioneer Male B group had the most accessions resistant to FER and GER (23 accessions). The other groups had similar resistance levels, with approximately 14 resistant accessions per group (Figure 6b).

**Figure 7:**
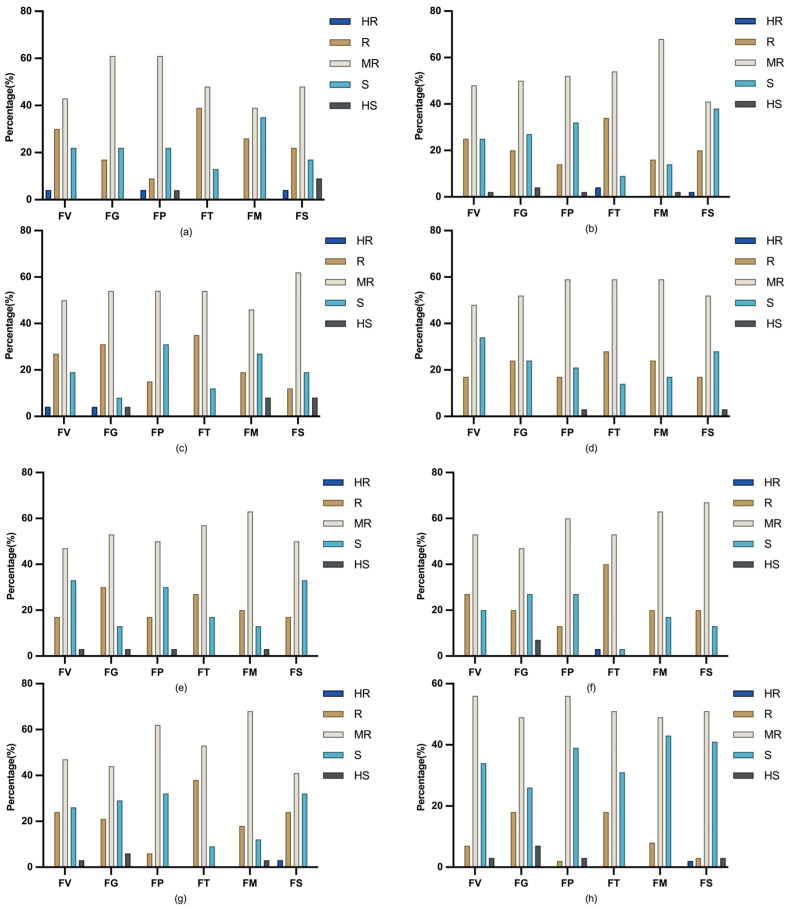
Distribution of resistance to six *Fusarium* species across heterotic groups. (**a**) Pioneer Male A group; (**b**) Pioneer Male B group; (**c**) SS group; (**d**) TSPT group; (**e**) PB group; (**f**) PH4CV group; (**g**) NSS group; (**h**) Pioneer Female group.

The Pioneer Male A group was most strongly resistant to ear rot caused by *F. temperatum* (with 87% of the tested accessions being moderately resistant or resistant), followed by ear rot caused by *F. subglutinans* and FER. This group exhibited partial resistance to ear rot caused by *F. proliferatum* and GER. However, it was poorly resistant to ear rot caused by *F. meridionale* (as 34% of the tested accessions were susceptible) (Figure 7a). The Pioneer Male B group was most resistant to ear rot caused by *F. temperatum* (85.7% of the tested accessions were moderately resistant or resistant), but it was also moderately resistant to FER and GER. However, its resistance was weakest for ear rot caused by *F. subglutinans* (where 37.5% of the tested accessions were susceptible) (Figure 7b). The SS group was most resistant to ear rot caused by *F. temperatum* (where 88.4% of the tested accessions were resistant or moderately resistant), followed by GER and ear rot caused by *F. subglutinans*, with intermediate resistance to FER and ear rot caused by *F. meridionale* or *F. proliferatum* (Figure 7c). The TSPT group was resistant or moderately resistant to four *Fusarium* spp. (with more than 75% resistant materials), but it also exhibited intermediate resistance to FER and ear rot caused by *F. subglutinans* (Figure 7d). The PB group was similarly resistant to GER and ear rot caused by *F. temperatum* or *F. meridionale* (where 83% of the tested accessions were moderately resistant or resistant), but 33% of the accessions in this group were susceptible to ear rot caused by *F. verticillioides*, *F. subglutinans*, or *F. proliferatum* (Figure 7e). The PH4CV group had the highest overall resistance, with 93%, 87%, 83%, and 80% of the tested accessions being moderately resistant or resistant to ear rot caused by *F. temperatum*, *F. subglutinans*, *F. meridionale*, and *F. verticillioides* (Figure 7f). By contrast, the NSS group was effectively resistant to only ear rot caused by *F. temperatum* (where 90% of the tested accessions were moderately resistant or resistant) or *F. meridionale* (87%), with 30% of the tested accessions being susceptible to ear rot caused by the other four *Fusarium* species (Figure 7g). The Pioneer Female group exhibited the poorest resistance, with 34–42% of the tested accessions susceptible to all six *Fusarium* species (Figure 7h).

A comprehensive evaluation revealed that PH4CV was the group that was most resistant to ear rot, followed by the Pioneer Male and SS groups, which exhibited moderate, but statistically significant, resistance. By contrast, the TSPT and PB groups had intermediate resistance profiles, whereas NSS and Pioneer Female were consistently the groups with the least effective resistance to all tested *Fusarium* species.

## 3. Discussion

Maize ear rot caused by fungal pathogens is one of the most significant threats to maize production [2,34]. It severely affects the yield, and the associated production of various toxins depending on the pathogens poses a risk to human and animal safety. Therefore, controlling maize ear rot is crucial in ensuring economic stability.

Although certain fungicide formulations may be useful in controlling ear rot under controlled conditions [35], the diversity of pathogens and the complexity of environmental factors in the field are major challenges to their widespread application. Enhancing the inherent disease resistance of maize remains the most effective and economical approach to protecting against ear rot. Both domestic and international studies have extensively focused on screening for germplasm resources resistant to FER and GER, resulting in the identification of numerous superior resistant germplasm resources [36,37]. However, there has been limited research on resistance to multiple ear rot pathogens. In the current study, 343 germplasm resources were precisely evaluated for resistance to ear rot caused by six *Fusarium* spp. over three years (2022–2024). Promising resources, critical for breeding broadly adaptive, multi-resistant maize varieties, were identified.

Maize ear rot resistance is a genetically complex, polygenic quantitative trait [38]. The evaluation of ear rot resistance is influenced by several factors, including the field planting methods, cultivation systems, geography, temperature, annual rainfall, and other pests and diseases. Consequently, there may be discrepancies in the results obtained from different locations and years [39,40]. The epidemiological significance of the six studied *Fusarium* species also varies across years. *F. graminearum* and *F. verticillioides* were consistently detected at high frequencies. In contrast, species such as *F. temperatum* and *F. meridionale* were more sporadic, likely influenced by specific climatic conditions. These fluctuations may partially explain the year-to-year variation in disease severity and genotype performance. Thus, the resistance observed in this study may reflect both broad-spectrum resistance and species-specific responses shaped by the annual pathogen composition.

In the present study, the correlation coefficients for the resistance of the tested accessions to six ear rot pathogens ranged from −0.01 to 0.21 in 2023; this range was significantly influenced by the climate conditions. Temperature and humidity play crucial roles in disease development. In 2023, the overall disease incidence remained relatively low because of high temperatures (>40 °C) and drought in July, which not only hindered disease development but also affected maize growth. The climate in 2022 and 2024 was more conducive to ear rot, resulting in relatively consistent correlation coefficients among the infections by the six *Fusarium* spp.

In 2022, the *r*-values for the resistance of the tested accessions to six ear rot pathogens ranged from 0.18 to 0.51. Excluding GER, the *r*-values for the resistance of the tested accessions to ear rot caused by the other pathogens exceeded 0.4 (i.e., moderate correlations). In 2024, the *r*-values for the resistance of the tested accessions to the six ear rot pathogens ranged from 0.36 to 0.71, with most *r*-values greater than 0.62 (i.e., strong correlations), including those for the resistance of the tested accessions to FER and ear rot caused by *F. proliferatum* or *F. subglutinans*. An analysis of the data compiled over the three-year study period revealed correlations between the resistance of the tested accessions to the six ear rot pathogens (*r*-values between 0.16 and 0.54), suggesting that certain materials might share similar ear rot resistance mechanisms, but this possibility will need to be experimentally validated. The correlations for the resistance of the tested accessions to other ear rot pathogens were relatively low. The response of each accession to different *Fusarium* spp. may vary significantly. According to previous research involving maize, resistance to FER is significantly correlated with resistance to GER [41,42]. In the current study, the *r*-values for the resistance to these two *Fusarium* species in 2022 and 2024 were 0.46 and 0.71, respectively, confirming a certain level of association. However, resistance correlations for the other *Fusarium* spp. have not been reported. This study detected moderate correlations in the resistance to ear rot caused by most of the tested pathogens, with the exception of resistance to ear rot caused by *F. proliferatum*, which was not highly correlated with resistance to ear rot caused by the other pathogens. These findings may need to be further validated in future studies.

**Figure 8:**
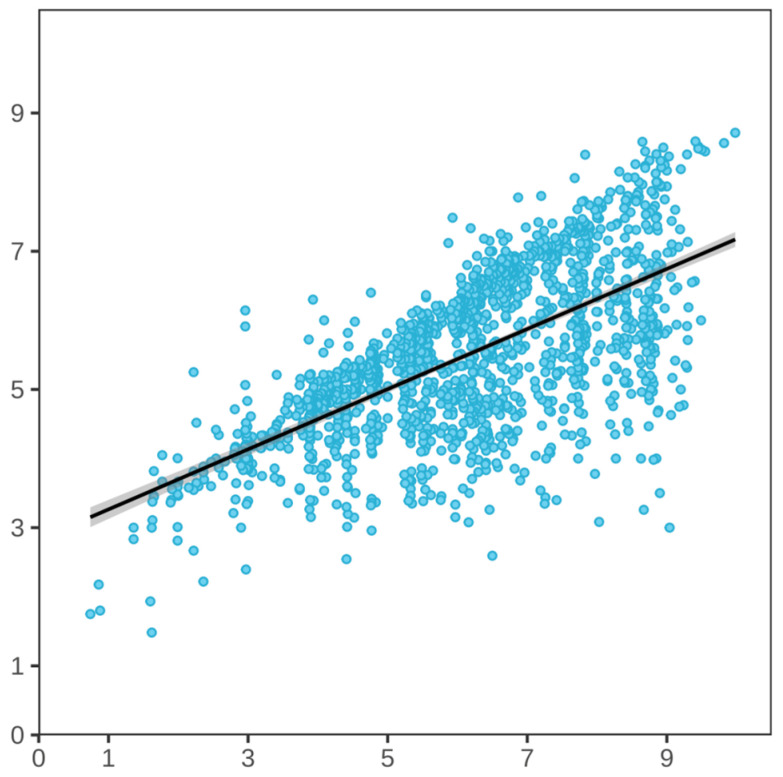
Scatter plot of visual and machine-assisted phenotyping for Fusarium ear rot. X-axis: machine-assisted survey, Y-axis: visual survey. Black line: y = 0.435x + 2.83.

Currently, the two main methods of evaluating maize ear rot are visual and machine-assisted surveys. Visual surveys remain the primary method for large-scale field evaluations [43]. Their advantages include their affordability, speed, and the fact that they can be conducted in all locations, but they are highly influenced by surveyor expertise and tend to have some inherent errors. Data from visual surveys typically represent the average disease scale across all ears of the same material, rather than the actual average diseased area. Although ears with 50% and 100% disease-affected areas are classified as highly susceptible (disease scale of 9), their actual resistance differs substantially. The correlation between the two methods was exceptionally strong (Pearson’s r = 0.65), indicating a moderately strong linear relationship. Machine-based phenotyping captured a broader phenotypic spectrum, demonstrating a superior resolution for extreme phenotypes—particularly in highly susceptible materials (Figure 8). Quantifying resistance levels on the basis of the mean disease-affected areas may be more precise than methods involving a conventional mean disease scale. Accordingly, the mean disease-affected area is crucial in quantifying the disease resistance of materials. Machine-assisted surveys rely on specialized imaging equipment and algorithms, thereby decreasing the need for expert knowledge among operators. These surveys produce consistent and precise results, eliminating human error, and are increasingly used for accurate ear rot resistance evaluations. However, issues regarding these surveys persist, including difficulties in distinguishing Fusarium ear rot lesions from other lesions (i.e., not caused by *Fusarium* spp.), which may lead to overestimations. Additionally, mechanical damage to kernels may lead to inflated readings. Continually optimizing algorithms is expected to lead to increased accuracy (i.e., data that match actual disease levels).

Survey methods should be selected according to specific needs. For the large-scale screening of materials in fields or assessments of varietal resistance where quantitative analyses of ear rot resistance are not required, visual surveys are clearly more suitable. However, for the precise gene mapping of ear rot resistance or accurate examinations of varietal resistance, machine surveys are more appropriate because of their accuracy and lack of human error. Future research should aim to apply machine learning and algorithms to screen for maize ear rot.

## 4. Materials and Methods

### 4.1. Test Materials

A total of 343 representative maize inbred lines with rich genetic diversity were used in this study. These lines were sourced from various institutions (e.g., Henan Academy of Agricultural Sciences, Heilongjiang Academy of Agricultural Sciences, Liaoning Academy of Agricultural Sciences, Beijing Academy of Agriculture and Forestry, Chinese Academy of Agricultural Sciences, Sichuan Agricultural University, and China Agricultural University). A list of the germplasm resources evaluated in this study is included in Table A1.

### 4.2. Pathogen and Inoculum Preparation

**Figure 9:**
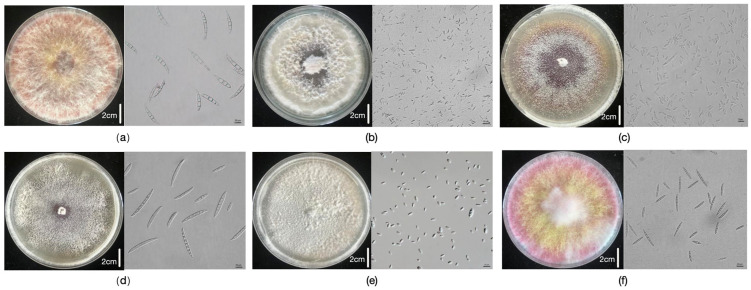
Colony and spore morphologies of six Fusarium spp. (**a**) *F. graminearum*; (**b**) *F. proliferatum*; (**c**) *F. subglutinans*; (**d**) *F. temperatum*; (**e**) *F. verticillioides*; (**f**) *F. meridionale*.

The six highly pathogenic *Fusarium* spp. (*F. verticillioides*, *F. graminearum*, *F. proliferatum*, *F. meridionale*, *F. subglutinans*, and *F. temperatum*) used in this experiment were isolated, identified, preserved, and cultured in our laboratory [14]. For inoculation, we used a single isolate per species. Figure 9 presents the colony and spore morphologies of these six isolates.

### 4.3. Disease Identification Plot

Maize resistance to ear rot was precisely evaluated in plots with environmental conditions conducive to disease development. These plots were located in the Changping (40°10′ N, 116°14′ E) and Shunyi (40°13′ N, 116°65′ E) districts of Beijing. The resistance of the germplasm resources was assessed in Changping in 2022–2023 and in both Changping and Shunyi in 2024. All materials were planted in mid-May consistently from 2022 to 2024. Experimental materials were randomly arranged, with each material planted in two rows (one row in Changping and one in Shunyi in 2024). Each row was 5.0 m long, with row spacing of 0.6 m and 25 plants per row. Susceptible and resistant control materials (B73 and X178, respectively) were included at every 100 rows. To prevent pollination disruption by pests, *Ostrinia furnacalis* and *Mythimna separata* were controlled with 5% emamectin benzoate spray during the seedling (V3) and bell (V12) stages, respectively.

### 4.4. Inoculum Preparation

Preparation of pea broth: A volume of 40 g/L dried peas was boiled in 1 L water for 30 min. The suspension was filtered through a cheesecloth, after which the volume was adjusted to 1 L, and it was autoclaved at 121 °C for 30 min.

Preparation of spore suspension: Six highly pathogenic *Fusarium* spp. activated from PDA medium in plates were cut into small pieces and added to sterile liquid pea broth. The culture was incubated at 25 °C in darkness, with shaking at 180 rpm, for 7–10 days. The mycelium was filtered and the spore suspension was collected and prepared for a concentration of 1 × 10^6^ spores/mL.

### 4.5. Inoculation Method

Duan et al. and Wang et al. compared the efficacy of two major inoculation methods, namely silk channel injection and ear injection (traumatic inoculation), for the evaluation of maize ear rot resistance; the results obtained from both methods were highly consistent (correlation coefficients > 0.90) [29,44]. The silk channel injection method, which more closely mimics the natural infection of maize ears by *Fusarium* spp., was selected for this study. At 3–5 days post-silking or when silks attained 5–10 cm in length, a calibrated continuous syringe was laterally inserted into the hollow central cavity of the silk channel. Each ear was inoculated with 2 mL of spore suspension via injection, with only the primary ear per plant being inoculated. Each type of ear rot was inoculated in two rows, with a total of 50 plants.

### 4.6. Disease Resistance Survey and Evaluation

Disease resistance was evaluated at the full maturity stage. For each accession, husk leaves were removed from the ears and the first ears were collected. Disease resistance was assessed using a machine-assisted system (maize ear rot image recognition and automated imaging system). The following describes the general working principles of the machine-assisted system used for disease evaluation. To ensure clear visualization, excess silks and husk leaves adhering to the maize ears were carefully removed prior to imaging. Each ear from the same genotype was sequentially placed into a machine equipped with rolling rollers and an overhead camera. Under computer control, the camera captured 12 evenly spaced images of each ear as it rotated within the rollers. A custom algorithm (the algorithm resembled that of “The Ear Unwrapper” [45] and the image system used by Wen et al. [46]) was then used to calculate the diseased area from the 12 images of each ear. The average diseased area of all ears from the same line was calculated to represent the disease severity of that line. The diseased area was then converted to a disease severity grade for an evaluation of resistance (Table 5). A visual survey was also conducted to generate supplementary data. Specifically, the diseased area was visually inspected and classified according to the percentage of the ear with disease symptoms. The average disease severity score was calculated and used for the final evaluation of resistance.

### 4.7. Statistical Analysis of Genotypic Variance and G × E Interaction

To assess the genetic variation and genotype-by-environment interactions for resistance to ear rot, a two-way analysis of variance (ANOVA) was conducted across three consecutive years for each *Fusarium* species. The model included the genotype, year, and their interaction as fixed effects. Genotypic variance and the proportion of variance explained by G × E interaction were estimated. The significance of genotype effects was tested using F-statistics (*p* < 0.05). The relative magnitude of the G × E interaction was calculated as the percentage of the total phenotypic variation attributed to the interaction term. All statistical analyses were performed using R (v4.2.0) or SAS (v9.4).

### 4.8. Heterotic Group Classification

A total of 294 maize inbred lines were genotyped by sequencing. The population structure was inferred using STRUCTURE v2.3.4, with the following settings [47]: admixture model and correlated allele frequencies; 50,000 iterations for Markov Chain Monte Carlo (MCMC) stabilization in the burn-in period; 100,000 iterations for MCMC replicates for the post-burn-in period. The number of clusters (K) was tested from 1 to 10, with 10 independent runs per K. The most probable number of genetic clusters (K) was identified using the ΔK method [48] implemented in STRUCTURE HARVESTER v0.6.94. Population structure bar plots were generated using Distruct v1.1, with individuals ordered according to Q-values (ancestry coefficients) [49,50]. A threshold of 0.65 was set to classify individuals into clusters after STRUCTURE analysis; individuals with Q-values greater than or equal to 0.65 were assigned to the corresponding group.

## 5. Conclusions

In this study, we evaluated the resistance of 343 maize germplasm resources to ear rot caused by six major *Fusarium* species over three consecutive years. A total of 69 and 77 lines were resistance to six and five ear rot diseases, respectively, while 139 lines were resistant to both FER and GER. These results highlight the presence of valuable broad-spectrum resistance within the tested germplasm. Furthermore, heterotic group analysis revealed significant variation in resistance, with the PH4CV group exhibiting the highest level of resistance. This work provides important genetic resources and insights for the breeding of maize cultivars with enhanced and durable ear rot resistance.

## Figures and Tables

**Table 1:** Geographic distribution and ecological dominance of *Fusarium* species in China.

*Fusarium* sp.	Geographic Distribution	Ecological Status
*F. verticillioides*	All primary maize agroecological zones	Dominant species
*F. graminearum*	All primary maize agroecological zones	Dominant species
*F. proliferatum*	Northeast, northwest, southwest	Regionally dominant species
*F. meridionale*	Southwest, southeast	Regionally dominant species
*F. subglutinans*	Northeast, northwest	Regionally dominant species
*F. temperatum*	Northeast	Regionally dominant species
*F. oxysporum*	Geographically undifferentiated distribution	Endemic species
*F. culmorum*	Geographically undifferentiated distribution	Endemic species
*F. solani*	Geographically undifferentiated distribution	Endemic species
*F. semitectum*	Geographically undifferentiated distribution	Endemic species
*F. fujikuroi*	Geographically undifferentiated distribution	Endemic species

**Table 2:** The mean disease severity for ear rot caused by six *Fusarium* species in three years.

	*F. verticillioides* Ear Rot	*F. graminearum* Ear Rot	*F. proliferatum* Ear Rot	*F. temperatum* Ear Rot	*F. meridionale* Ear Rot	*F. subglutinans* Ear Rot
2022	5.76	5.28	5.86	5.47	5.79	6.07
2023	3.56	3.61	4.53	3.03	3.69	4.24
2024	6.55	6.59	6.26	6.10	6.89	6.36

**Table 3:** Significance of genotypic variance and heritability values among six Fusarium ear rot types.

Fusarium Ear Rot	Genotypic Variance	F-Value	Broad Sense Heritability Value	*p*-Value
*F. graminearum* ear rot	1.85 ± 0.21	24.37	0.86	<0.001, ***
*F. verticillioides* ear rot	1.62 ± 0.18	19.83	0.83	<0.001, ***
*F. proliferatum* ear rot	1.28 ± 0.15	15.02	0.54	<0.001, ***
*F. temperatum* ear rot	0.97 ± 0.11	10.45	0.32	0.003, **
*F. meridionale* ear rot	0.89 ± 0.10	9.12	0.31	0.008, **
*F. subglutinans* ear rot	0.75 ± 0.09	7.85	0.29	0.021, *

Data markers indicate statistical significance (*: *p* < 0.05; **: *p* < 0.01; ***: *p* < 0.001).

**Table 4:** Contribution of genotype × environment interaction to phenotypic variance in ear rot caused by six *Fusarium* species.

Fusarium Ear Rot	G × E Interaction Variance	Percentage of Total Variance
*F. graminearum* ear rot	0.38 ± 0.05	17.2%
*F. verticillioides* ear rot	0.42 ± 0.06	20.6%
*F. proliferatum* ear rot	0.61 ± 0.08	32.3%
*F. temperatum* ear rot	0.75 ± 0.09	43.6%
*F. meridionale* ear rot	0.68 ± 0.08	43.3%
*F. subglutinans* ear rot	0.59 ± 0.07	44.0%

**Table 5:** Classification of disease severity levels and criteria for evaluation of ear rot resistance.

Scale	Description	Ear Rot Score	Resistance
1	0~1% of the diseased ear surface	≤1.5	Highly resistant (HR)
3	2~10% of the diseased ear surface	1.6~3.5	Resistant (R)
5	11~25% of the diseased ear surface	3.6~5.5	Moderately resistant (MR)
7	26~50% of the diseased ear surface	5.6~7.5	Susceptible (S)
9	51~100% of the diseased ear surface	7.6~9.0	Highly susceptible (HS)

## Data Availability

The datasets generated and/or analyzed during the current study are available from the corresponding author on reasonable request.

## Appendix A

**Table A1:** Codes and heterotic groups of maize inbred lines used in this study.

Code	Heterotic Group	Code	Heterotic Group	Code	Heterotic Group
K21HZD0021	NSS	K21HZD1517	Pioneer Female	K21HZD5457	Pioneer Male B
K21HZD0044	NSS	K21HZD1548	Pioneer Female	K21HZD5461	Pioneer Male B
K21HZD0071	NSS	K21HZD1550	Pioneer Female	K21HZD5970	Pioneer Male B
K21HZD0187	NSS	K21HZD1558	Pioneer Female	K21HZD5973	Pioneer Male B
K21HZD0271	NSS	K21HZD1978	Pioneer Female	K21HZD5976	Pioneer Male B
K21HZD0275	NSS	K21HZD1983	Pioneer Female	K21HZD6054	Pioneer Male B
K21HZD0375	NSS	K21HZD2118	Pioneer Female	K21HZD6057	Pioneer Male B
K21HZD0394	NSS	K21HZD2123	Pioneer Female	K21HZD6063	Pioneer Male B
K21HZD0395	NSS	K21HZD2124	Pioneer Female	K21HZD0012	SS
K21HZD0401	NSS	K21HZD2130	Pioneer Female	K21HZD0105	SS
K21HZD0402	NSS	K21HZD2131	Pioneer Female	K21HZD0452	SS
K21HZD0482	NSS	K21HZD2228	Pioneer Female	K21HZD0611	SS
K21HZD0566	NSS	K21HZD2239	Pioneer Female	K21HZD0700	SS
K21HZD0751	NSS	K21HZD2253	Pioneer Female	K21HZD0736	SS
K21HZD0759	NSS	K21HZD2258	Pioneer Female	K21HZD0762	SS
K21HZD0766	NSS	K21HZD2278	Pioneer Female	K21HZD1623	SS
K21HZD0777	NSS	K21HZD2357	Pioneer Female	K21HZD1693	SS
K21HZD0792	NSS	K21HZD2365	Pioneer Female	K21HZD1786	SS
K21HZD0847	NSS	K21HZD2415	Pioneer Female	K21HZD1971	SS
K21HZD0850	NSS	K21HZD2588	Pioneer Female	K21HZD1985	SS
K21HZD1131	NSS	K21HZD2598	Pioneer Female	K21HZD2127	SS
K21HZD1133	NSS	K21HZD4687	Pioneer Female	K21HZD2146	SS
K21HZD1145	NSS	K21HZD4698	Pioneer Female	K21HZD2199	SS
K21HZD1172	NSS	K21HZD4705	Pioneer Female	K21HZD2388	SS
K21HZD1214	NSS	K21HZD4731	Pioneer Female	K21HZD2405	SS
K21HZD1463	NSS	K21HZD4757	Pioneer Female	K21HZD2442	SS
K21HZD1465	NSS	K21HZD5217	Pioneer Female	K21HZD2612	SS
K21HZD1467	NSS	K21HZD5267	Pioneer Female	K21HZD3969	SS
K21HZD1979	NSS	K21HZD5274	Pioneer Female	K21HZD4182	SS
K21HZD2550	NSS	K21HZD5279	Pioneer Female	K21HZD4303	SS
K21HZD4741	NSS	K21HZD5290	Pioneer Female	K21HZD4790	SS
K21HZD5318	NSS	K21HZD5291	Pioneer Female	K21HZD5082	SS
K21HZD5376	NSS	K21HZD5375	Pioneer Female	K21HZD5296	SS
K21HZD5988	NSS	K21HZD5946	Pioneer Female	K21HZD5454	SS
K21HZD0010	PB	K21HZD5949	Pioneer Female	K21HZD5650	SS
K21HZD0018	PB	K21HZD5952	Pioneer Female	K21HZD0051	TSPT
K21HZD0334	PB	K21HZD5955	Pioneer Female	K21HZD0103	TSPT
K21HZD0471	PB	K21HZD5958	Pioneer Female	K21HZD0761	TSPT
K21HZD0569	PB	K21HZD5991	Pioneer Female	K21HZD0844	TSPT
K21HZD0715	PB	K21HZD6006	Pioneer Female	K21HZD0871	TSPT
K21HZD0897	PB	K21HZD6018	Pioneer Female	K21HZD1179	TSPT
K21HZD1854	PB	K21HZD6021	Pioneer Female	K21HZD1380	TSPT
K21HZD1948	PB	K21HZD6027	Pioneer Female	K21HZD1410	TSPT
K21HZD1967	PB	K21HZD0074	Pioneer Male A	K21HZD1429	TSPT
K21HZD1993	PB	K21HZD0095	Pioneer Male A	K21HZD1485	TSPT
K21HZD2220	PB	K21HZD0224	Pioneer Male A	K21HZD1499	TSPT
K21HZD2417	PB	K21HZD0236	Pioneer Male A	K21HZD1634	TSPT
K21HZD2473	PB	K21HZD0279	Pioneer Male A	K21HZD2154	TSPT
K21HZD2475	PB	K21HZD0341	Pioneer Male A	K21HZD2177	TSPT
K21HZD2595	PB	K21HZD0397	Pioneer Male A	K21HZD2243	TSPT
K21HZD2596	PB	K21HZD0398	Pioneer Male A	K21HZD2423	TSPT
K21HZD2779	PB	K21HZD0444	Pioneer Male A	K21HZD2425	TSPT
K21HZD2817	PB	K21HZD0511	Pioneer Male A	K21HZD2429	TSPT
K21HZD2839	PB	K21HZD0562	Pioneer Male A	K21HZD2439	TSPT
K21HZD2844	PB	K21HZD0691	Pioneer Male A	K21HZD2509	TSPT
K21HZD2879	PB	K21HZD0731	Pioneer Male A	K21HZD2555	TSPT
K21HZD3076	PB	K21HZD1027	Pioneer Male A	K21HZD2556	TSPT
K21HZD3156	PB	K21HZD1132	Pioneer Male A	K21HZD3945	TSPT
K21HZD3435	PB	K21HZD1155	Pioneer Male A	K21HZD5342	TSPT
K21HZD4242	PB	K21HZD1344	Pioneer Male A	K21HZD5629	TSPT
K21HZD4690	PB	K21HZD1970	Pioneer Male A	K21HZD5961	TSPT
K21HZD5166	PB	K21HZD2373	Pioneer Male A	K21HZD5964	TSPT
K21HZD5459	PB	K21HZD2374	Pioneer Male A	K21HZD5967	TSPT
K21HZD5982	PB	K21HZD2376	Pioneer Male A	K21HZD5979	TSPT
K21HZD5985	PB	K21HZD2377	Pioneer Male A	K21HZD6042	TSPT
K21HZD0057	PH4CV	K21HZD4780	Pioneer Male A	K21HZD6048	TSPT
K21HZD0171	PH4CV	K21HZD0025	Pioneer Male B	2021S0915	
K21HZD0196	PH4CV	K21HZD0068	Pioneer Male B	2021S0919	
K21HZD0197	PH4CV	K21HZD0072	Pioneer Male B	2021S0925	
K21HZD0210	PH4CV	K21HZD0214	Pioneer Male B	2021S0926	
K21HZD0390	PH4CV	K21HZD0512	Pioneer Male B	2021S0928	
K21HZD0415	PH4CV	K21HZD0526	Pioneer Male B	2021S0929	
K21HZD0749	PH4CV	K21HZD0730	Pioneer Male B	2021S0931	
K21HZD0904	PH4CV	K21HZD0836	Pioneer Male B	2021S0933	
K21HZD1193	PH4CV	K21HZD0842	Pioneer Male B	2021S0935	
K21HZD1205	PH4CV	K21HZD0852	Pioneer Male B	2021S0940	
K21HZD1208	PH4CV	K21HZD0857	Pioneer Male B	2021S0941	
K21HZD1210	PH4CV	K21HZD0866	Pioneer Male B	2021S0943	
K21HZD1237	PH4CV	K21HZD0868	Pioneer Male B	2021S0945	
K21HZD1245	PH4CV	K21HZD0885	Pioneer Male B	2021S0960	
K21HZD1514	PH4CV	K21HZD0898	Pioneer Male B	2021S0961	
K21HZD1526	PH4CV	K21HZD0899	Pioneer Male B	22NHRB001	
K21HZD1617	PH4CV	K21HZD0903	Pioneer Male B	22NHRB016	
K21HZD1636	PH4CV	K21HZD1149	Pioneer Male B	22NHRB039	
K21HZD1637	PH4CV	K21HZD1182	Pioneer Male B	22NHRB048	
K21HZD1640	PH4CV	K21HZD1183	Pioneer Male B	22NHRB066	
K21HZD1944	PH4CV	K21HZD1200	Pioneer Male B	22NHRB081	
K21HZD1987	PH4CV	K21HZD1217	Pioneer Male B	22NHRB105	
K21HZD2300	PH4CV	K21HZD1520	Pioneer Male B	22NHRB108	
K21HZD2567	PH4CV	K21HZD1573	Pioneer Male B	22NHRB155	
K21HZD2578	PH4CV	K21HZD1581	Pioneer Male B	22NHRB214	
K21HZD2581	PH4CV	K21HZD1590	Pioneer Male B	22NHRB315	
K21HZD2602	PH4CV	K21HZD1598	Pioneer Male B	22NHRB322	
K21HZD2610	PH4CV	K21HZD1615	Pioneer Male B	CNH3323	
K21HZD4732	PH4CV	K21HZD1962	Pioneer Male B	CY103	
K21HZD0170	Pioneer Female	K21HZD1965	Pioneer Male B	CY105	
K21HZD0226	Pioneer Female	K21HZD2234	Pioneer Male B	CY109	
K21HZD0296	Pioneer Female	K21HZD2244	Pioneer Male B	K21HXY045	
K21HZD0400	Pioneer Female	K21HZD2260	Pioneer Male B	K21HXY085	
K21HZD0742	Pioneer Female	K21HZD2274	Pioneer Male B	K21HXY145	
K21HZD0786	Pioneer Female	K21HZD2350	Pioneer Male B	K21HXY221	
K21HZD0804	Pioneer Female	K21HZD2371	Pioneer Male B	K21HXY259	
K21HZD0849	Pioneer Female	K21HZD2398	Pioneer Male B	K21HZD0276	
K21HZD0861	Pioneer Female	K21HZD2463	Pioneer Male B	K21HZD0887	
K21HZD0915	Pioneer Female	K21HZD2482	Pioneer Male B	K21HZD1353	
K21HZD1261	Pioneer Female	K21HZD2534	Pioneer Male B	K21HZD1470	
K21HZD1295	Pioneer Female	K21HZD2553	Pioneer Male B	K21HZD1521	
K21HZD1302	Pioneer Female	K21HZD2616	Pioneer Male B	K21HZD1528	
K21HZD1307	Pioneer Female	K21HZD2618	Pioneer Male B	K21HZD2219	
K21HZD1309	Pioneer Female	K21HZD3093	Pioneer Male B	K21HZD4411	
K21HZD1323	Pioneer Female	K21HZD4339	Pioneer Male B	K21HZD4653	
K21HZD1370	Pioneer Female	K21HZD4737	Pioneer Male B	K21HZD4739	
K21HZD1418	Pioneer Female	K21HZD5309	Pioneer Male B	K21HZD5266	
K21HZD1511	Pioneer Female	K21HZD5366	Pioneer Male B	K21HZD5308	
				K21HZD5467	
